# Time synchronisation for millisecond-precision on bio-loggers

**DOI:** 10.1186/s40462-024-00512-7

**Published:** 2024-10-28

**Authors:** Timm A. Wild, Georg Wilbs, Dina K. N. Dechmann, Jenna E. Kohles, Nils Linek, Sierra Mattingly, Nina Richter, Spyros Sfenthourakis, Haris Nicolaou, Elena Erotokritou, Martin Wikelski

**Affiliations:** 1 Department of Migration, Max Planck Institute of Animal Behavior, 78315 Radolfzell, Germany; 2 Department of Biology, University of Konstanz, 78464 Konstanz, Germany; 3 Department of Biological Sciences, University of Cyprus, Nicosia, 1678 Cyprus; 4 Rural Development and Environment, Ministry of Agriculture, 2025 Strovolos Nicosia, Nicosia, Cyprus

**Keywords:** Animal tracking, Movement ecology, Telemetry, Wireless sensors, Embedded systems, WiFi, GPS, Real time, Proximity, Internet of animals, IoT

## Abstract

Time-synchronised data streams from bio-loggers are becoming increasingly important for analysing and interpreting intricate animal behaviour including split-second decision making, group dynamics, and collective responses to environmental conditions. With the increased use of AI-based approaches for behaviour classification, time synchronisation between recording systems is becoming an essential challenge. Current solutions in bio-logging rely on manually removing time errors during post processing, which is complex and typically does not achieve sub-second timing accuracies.

We first introduce an error model to quantify time errors, then optimise three wireless methods for automated onboard time (re)synchronisation on bio-loggers (GPS, WiFi, proximity messages). The methods can be combined as required and, when coupled with a state-of-the-art real time clock, facilitate accurate time annotations for all types of bio-logging data without need for post processing. We analyse time accuracy of our optimised methods in stationary tests and in a case study on 99 Egyptian fruit bats (*Rousettus aegyptiacus*). Based on the results, we offer recommendations for projects that require high time synchrony.

During stationary tests, our low power synchronisation methods achieved median time accuracies of 2.72 / 0.43 ms (GPS / WiFi), compared to UTC time, and relative median time accuracies of 5 ms between tags (wireless proximity messages). In our case study with bats, we achieved a median relative time accuracy of 40 ms between tags throughout the entire 10-day duration of tag deployment. Using only one automated resynchronisation per day, permanent UTC time accuracies of ≤ 185 ms can be guaranteed in 95% of cases over a wide temperature range between 0 and 50 °C. Accurate timekeeping required a minimal battery capacity, operating in the nano- to microwatt range.

Time measurements on bio-loggers, similar to other forms of sensor-derived data, are prone to errors and so far received little scientific attention. Our combinable methods offer a means to quantify time errors and autonomously correct them at the source (i.e., on bio-loggers). This approach facilitates sub-second comparisons of simultaneously recorded time series data across multiple individuals and off-animal devices such as cameras or weather stations. Through automated resynchronisations on bio-loggers, long-term sub-second accurate timestamps become feasible, even for life-time studies on animals. We contend that our methods have potential to greatly enhance the quality of ecological data, thereby improving scientific conclusions.

## Background

Bio-loggers are animal-borne devices that collect ecologically relevant sensor data, including but not limited to data from GPS, accelerometers, barometers, microphones, and thermometers [1]. These devices recently opened up new directions in wildlife research, helping to understand a wide range of phenomena such as long-distance disease transmissions by migratory birds [2] or the flight path control of migrating moths [3]. Recent technological innovations, including the internet of animals [4] or low cost open source tag designs [5–9], allow ecologists to collect data from more individuals with increased resolutions over prolonged durations. Most gathered datasets are analysed as time series, contingent upon precise time annotations. Such time annotations (e.g., timestamps in UTC format), are critical to understand split-second decision making [10], group dynamics [11], responses to weather conditions [12], behaviour synchronisation [13] or spatial-temporal interactions [14] of animals.

Time measurements allow the comparison of data from tagged animals with external measurements, events, and other simultaneously tagged individuals. For example, time measurements have permitted the study of the timing of migration departure relative to wind and rain conditions for individual robins [12] or the responses of snowshoe hares to moonlight intensity [15]. For some behaviours, milliseconds matter, such as interactions between predators and prey [16, 17]. Synchronised data from multiple tags have been used to, for example, track sickness effects on the behaviour of groups of wild vampire bats [18]. Machine learning algorithms based on fine-scale sensor data are often used to classify behaviours [19–22]. Annotated training data for such AI algorithms often require high temporal synchronisation between multiple data sources such as video recordings and animal-borne accelerometers [20]. Additionally, attaching several tags to different parts of the same individual requires close temporal synchronisation to match fine-scale movements [23, 24]. Time synchronisation also helps to reduce the high energetic cost of wireless communication on bio-loggers by reducing reception time windows, as seen in proximity networks [25–27]. Furthermore, precise timing allows devices to trigger events at specific times, including data downloads during a pass of an unmanned aerial vehicle [11, 28] or satellite [29]. While there is strong scientific focus on quantifying spatial accuracy on bio-logging devices (e.g., from GPS data [30]), temporal accuracy has received far less attention, despite being frequently mentioned as a primary limitation [11, 31–33]. The quality and power of ecological conclusions drawn from multi-source and multi-sensor data depends on the accuracy of time measurements.

Time, like any other sensor measurement, is susceptible to inaccuracies due to the inherent limitations of clocks [34, 35]. Accumulated clock errors led to serious problems with the communication and navigation systems in other fields such as of a Mars exploration rover [36]. Compared to stationary systems, measuring time on bio-loggers is much more challenging due to weight, size and energy limitations. Within bio-loggers, time is measured using GPS time references [30], network time protocol (NTP) data obtained via WiFi [37], internal clocks, or a combination of these. GPS allows a theoretical time accuracy in the range of nanoseconds [38], but additional errors are introduced by data processing, communication interfaces and clock drifts between fixes. For example, a 2019 study found average discrepancies of up to 28 s between acceleration and GPS data recorded on the same device after nine days [39]. Using GPS for time requires additional hardware, consumes substantial battery power, and falters where satellites are obstructed. The integration of WiFi and NTP on bio-loggers is a new concept that has shown promising accuracies below ± 100 ms but requires WiFi infrastructure [37]. Time measurements can also be performed offboard, with devices that implement wireless communication where receiving stations can measure UTC time, e.g., with internet of things (IoT) technologies such as Sigfox [40] or LoRa [41]. However, with these technologies, accuracies are often unspecified and sub-second resolutions are uncommon. Many micro-sized bio-loggers do not offer any onboard time synchronisation and use only oscillators of microcontrollers, sensors and/or real time clock (RTC) modules for measuring time, e.g., IMU loggers [42], barometric pressure loggers [9], or miniaturised temperature loggers [43]. When using bio-loggers without onboard time synchronisation, ecologists often annotate data with UTC time by looking at their watches when starting and/or stopping measurements, by estimating daily patterns based on light and/or temperature readings [44], or by creating unique data patterns that can be used as time reference in post processing (e.g., creating unique movements [45] or playing a unique sound [33]). Time synchronisations in post processing (e.g., between acceleration data and video data [32, 46], or acceleration data and audio data [47]) are complex, error prone and often assume a linear time drift. Time drift of electronic clocks is non-linear and varies with environmental conditions, including temperature and applied voltage, which can have major implications for battery-powered animal-borne devices exposed to severe environmental fluctuations (e.g., on Arctic foxes where habitat temperatures can drop as low as -45 °C [48]). The quality of ecological data is compromised by the accumulation of time errors, particularly when animals are tracked over long periods of time or entire lifespans [49].

To tackle these problems, we first developed a model to describe technical limitations of measuring time on bio-logging devices. We then evaluated three methods for wireless onboard time (re)synchronisations (GPS, WiFi, wireless proximity messages) and measured time accuracies and power consumption in off-animal experiments. Between synchronisation events we used the best-in-class temperature-compensated RTC to store time with reduced drift. Following the off-animal evaluation, we conducted a field study on 99 Egyptian fruit bats (*Rousettus aegyptiacus*) and showed how our onboard methods can be combined to achieve sub-40 ms time accuracies between devices over the course of 10 days. The results demonstrated that our methods enable the annotation any type of short- and long-term ecological data, including combined data streams (e.g., from GPS sensors, proximity sensors, microphones, accelerometers, magnetometers, thermometers, barometers) with highly accurate timestamps. By reducing time errors on devices, at the data source, complex synchronisation methods in post processing are becoming obsolete. The time accuracy of bio-loggers is gaining a key role for the exponentially growing number of studies that rely on the sub-second synchronicity of multiple data streams over extended periods of time.

## Methods

### Error model for measuring time on bio-loggers

Bio-loggers are battery-powered devices that derive time by accumulating ticks from electronic oscillators. Modern bio-loggers integrate microcontrollers that require such oscillators to execute program code. The same oscillators are also often used for generating time steps in sensor data. Most bio-loggers integrate multiple independent oscillators (e.g., inside microcontrollers, sensors, radio chips and RTC modules). RTCs are clocks that count physical time (e.g., in seconds, minutes and/or hours) [34]. In contrast, the term “real time” in the literature describes either devices that react in guaranteed times [50], devices that make collected data available with little delay [51, 52], or devices that measure time in a time zone format (e.g., UTC) [53]. Bio-loggers with GPS often annotate data with UTC time, since GPS satellites contain atomic clocks and propagate the current UTC time in their messages. When bio-loggers have access to the internet (e.g., via WiFi), UTC time can be obtained by querying an NTP server that distributes time from atomic clocks. Bio-loggers without GPS and WiFi often simply start counting time from zero when they are activated. To derive UTC time from such devices, scientists normally note the time of activation and add it to the relative time counter during post processing, which is error prone and limited by human response times. Alternatively, unique or repeating events that become visible in data can be used for time (re)synchronisation (e.g., special sounds that are recorded with audio tags, or changes in day lengths measured with light sensors [33, 44]).

**Fig. 1:**
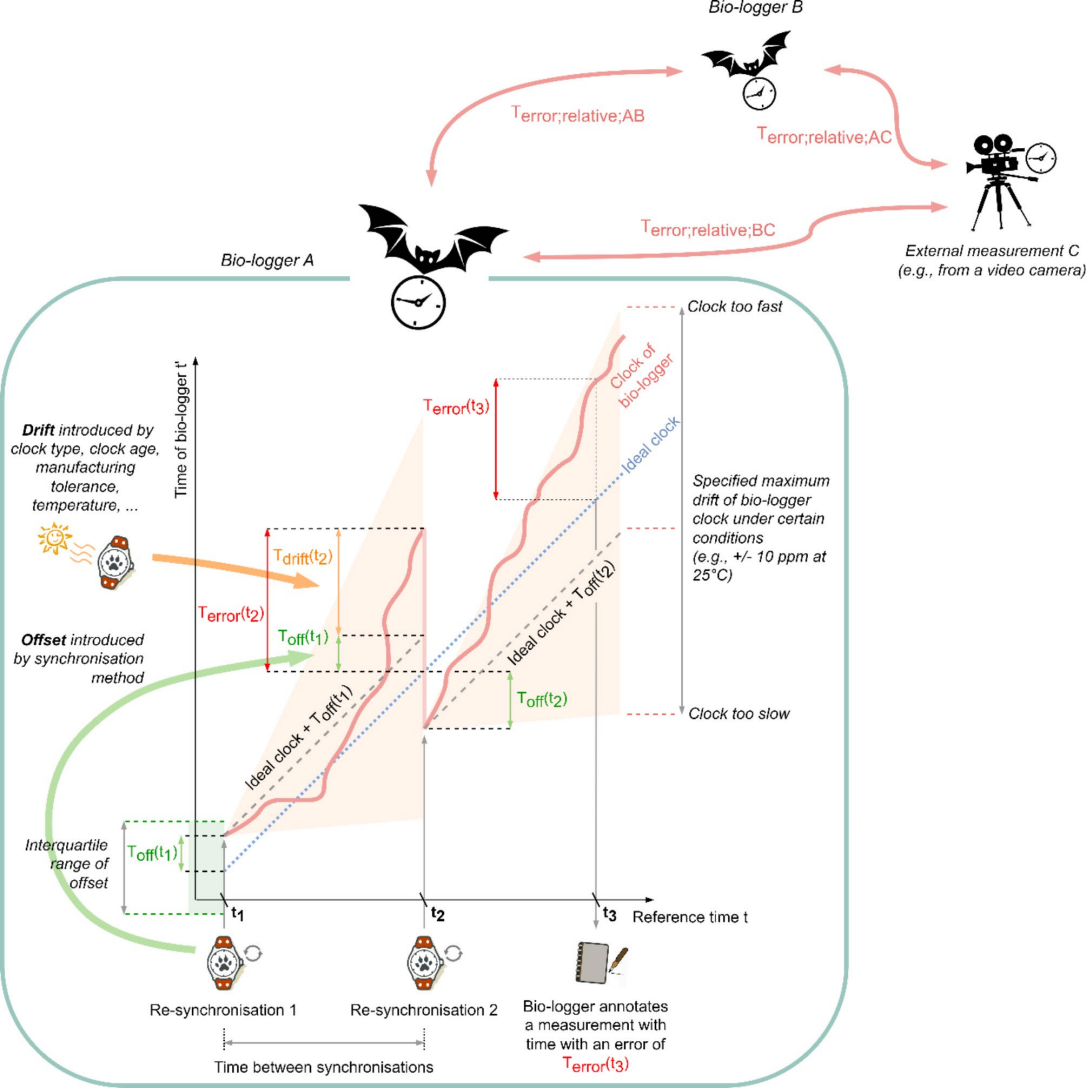
Error model for measuring time on bio-loggers. Drifts (*T*_*drift*_) and time offset errors (*T*_*off*_) of clocks integrated into bio-loggers lead to deviations from the reference time *t*. Different clocks have different errors, resulting in relative time differences (*T*_*error; relative*_) between devices

Here we define UTC time as reference time *t*. The time error *T*_*error*_*(t)* describes the deviation to the reference time *t* (Fig. 1). In our model *T*_*error*_*(t)* consists of two parts: *T*_*off*_*(t)* is the error that is introduced by a synchronisation method and that causes a constant offset to the reference time *t*. *T*_*off*_*(t)* does not differ between tags with the same hardware and firmware. *T*_*drift*_*(t)* is the error that is introduced by inaccuracies of ticking oscillators and varies between individual devices. Time *t’* is measured on the bio-logger and can be modelled as shown in Eq. (1).

1 $$\:{t}^{{\prime\:}}\left(t\right)=t+{T}_{error}\left(t\right)=t+{T}_{drift}\left(t\right)+{T}_{off}\left(t\right)$$

Electronic clocks are normally specified with a maximum drift expressed in parts per million (ppm) or percent (under certain conditions, e.g., temperature ranges), which then causes *T*_*drift*_ to grow over time (Table 1). *T*_*drift*_ applies also to clocks in sensors, meaning that, for example, an accelerometer configured to record data at 25 Hz will not produce exactly 25 samples per second. Each free-running sensor is drifting independently from other clocks on a bio-logger. It is important to mention that *T*_*drift*_ is not growing at a fixed rate, but in a highly non-deterministic way, influenced by factors such as ambient temperature, age of the unit, manufacturing tolerances and applied voltages. Synchronising time at the start and end of an experiment and assuming a linear time drift in post processing is a simplification that might result in inaccurate timestamps, especially during long-term experiments. When deploying multiple devices, each device drifts differently, leading to relative time differences (*T*_*error; relative*_) that complicate data comparison. To increase the accuracy of the measured time *t’* on a bio-logging device, it is necessary to combine methods that reduce both *T*_*off*_ and *T*_*drift*_.

**Table 1:** Examples of the effect of time drift on time-annotated data on bio-loggers. (*) the actual drift can be significantly higher when considering ambient temperature ranges, age of the unit, manufacturing tolerances and applied voltages. (**) due to the module size and power consumption, integration into bio-logging devices is not realistic

Example component	Rated maximum drift (ppm) (*)	Maximum T_drift_ per day (s)	Maximum T_drift_ per week (s)	Maximum T_drift_ per month (s)
Drift of an oven-controlled oscillator (OCXO) module between 0 and 50 °C (Abracon AOCJY, 25.4 × 22.1 × 12.7 mm) (**)	± 0.005	± 0.0004	± 0.0030	± 0.0134
Drift of the currently best temperature-compensated oscillator (TCXO) module between 0 and 50 °C (Micro Crystal RV-8803-C7, 3.2 × 1.5 × 0.8 mm)	± 1.5	± 0.13	± 0.9	± 4.0
Drift of the currently best temperature-compensated oscillator (TCXO) module between − 40 and 85 °C (Micro Crystal RV-8803-C7, 3.2 × 1.5 × 0.8 mm)	± 3	± 0.26	± 1.8	± 8.0
Drift of a high-precision 80 MHz oscillator of a common microcontroller at 25 °C (Espressif ESP32)	± 10	± 0.86	± 6.0	± 26.8
Drift of a non-temperature-compensated oscillator (XO) module between 0 and 50 °C (Micro Crystal RV-3028-C7, 3.2 × 1.5 × 0.8 mm)	± 20	± 1.73	± 12.1	± 53.6
Drift of the internal oscillator of a common microcontroller at 25 °C (Microchip ATmega328P)	± 20,000	± 1,728	± 12,069	± 53,568
Drift of the internal oscillator of a common microcontroller between − 40 and 125 °C (Microchip ATmega328P)	± 140,000	± 12,096	± 84,672	± 374,976

### Description of the WildFi Bio-logger

The WildFi bio-logger is a modular open source wildlife tracking platform that collects and wirelessly transmits data streams from GPS sensors, proximity sensors, accelerometers, magnetometers, gyroscopes, barometers, thermometers and humidity sensors [37]. The base tag (17.85 × 25.95 × 3.15 mm) can be expanded with add-on boards, facilitating the integration of additional sensors. The base tag integrates an ESP32 microcontroller capable of communicating via WiFi and Bluetooth. UTC time can be obtained via GPS or WiFi (NTP). To use GPS, an add-on board needs to be connected to the base tag. Time is stored on a temperature-compensated RTC (Micro Crystal RV-8803-C7). The RTC has a maximum drift specification of ± 3 ppm between − 40 and 85 °C, a resolution of 10 ms and a power consumption of 720 nW. Furthermore, the RTC can generate interrupts at particular times of the day (e.g., every day at 1:00 pm) to wake up the microcontroller and perform actions (e.g., sample data or initiate a wireless data transmission). Since both software and hardware are open source, scientists can write their own tag software in C++. We extended the existing open source firmware (github.com/trichl/WildFiOpenSource [54]) to improve the time synchronisation performance.

### Optimised methods for onboard wireless time (re)synchronisation

**Fig. 2:**
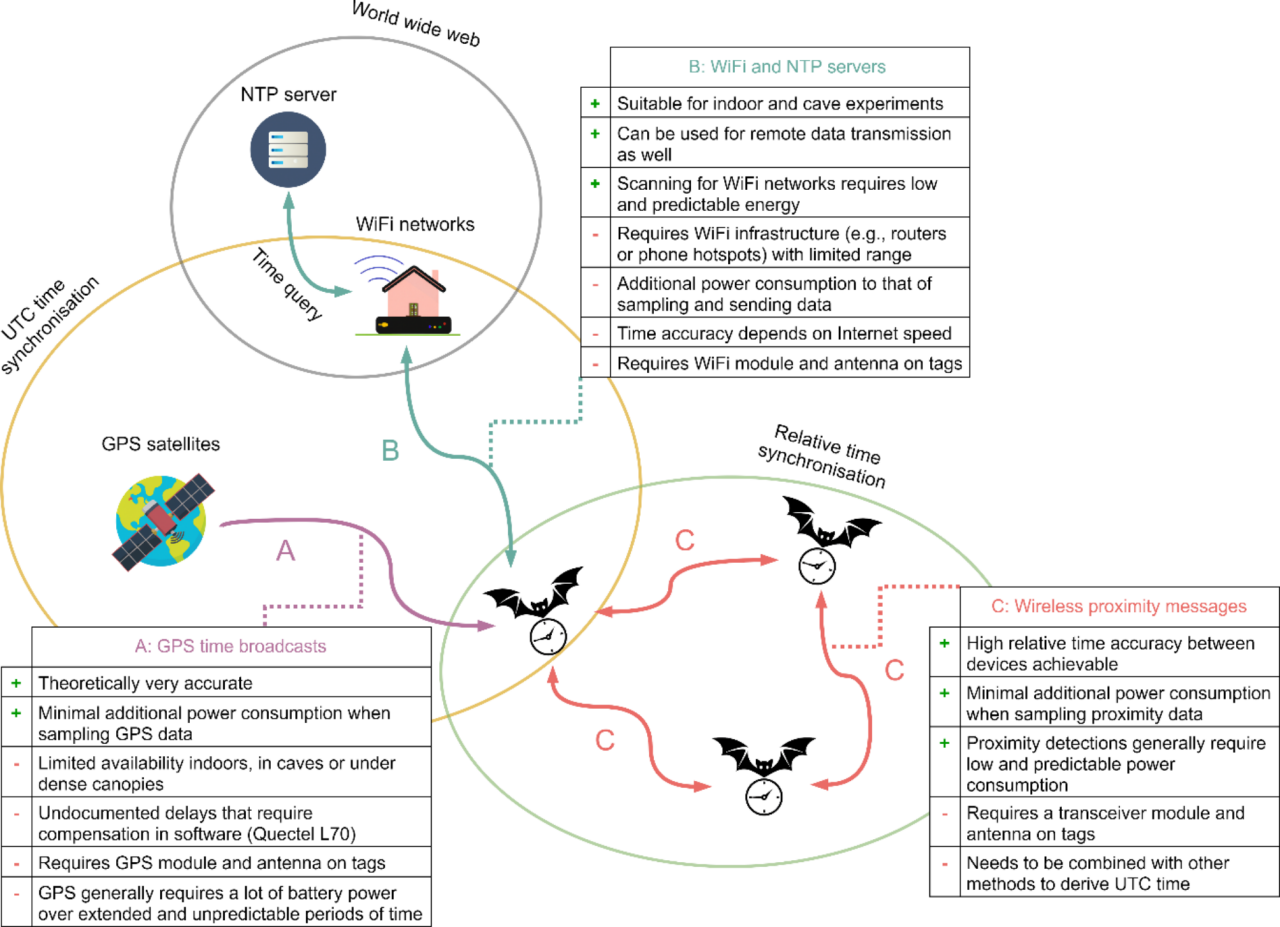
Overview of the three evaluated methods (GPS [**A**], WiFi and NTP servers [**B**], wireless proximity messages [**C**]) to automatically (re)synchronise time onboard bio-loggers

We integrated three wireless methods (A - C) for onboard time (re)synchronisation on the WildFi tags (Fig. 2). The methods can be combined depending on the tag and study design. For each method, we conducted stationary experiments to estimate the errors *T*_*off; GPS*_, *T*_*off; WiFi*_, and *T*_*error; relative*_. The stationary experiments were carried out with a total of two tags, for which we developed a special firmware for test automation.

### Method A: using GPS time broadcasts for onboard UTC time (re)synchronisation

For synchronisation method A (Fig. 2A), we used a WildFi tag with a GPS add-on board (GPS module [Quectel L70B-M39] and GPS antenna [Antenova SR4G013]). GPS time retrieval relies on establishing contact between tags and satellites and may fail when lacking a direct line of sight with at least one satellite. As is common with most tags, we used the hot start functionality of the GPS module. We extracted the time information from NMEA sentences that are transmitted via UART at 115,200 baud to the ESP32 microcontroller. We found that time is only reliably updated on the GPS module after getting a fix and therefore programmed the devices to first wait for a fix before updating the RTC. Furthermore, we found a time delay caused by the UART transmission of the GPS module to the microcontroller, and a time delay caused by the internal calculation speed of the GPS module that correlated with the number of visible satellites. We implemented an onboard compensation function for these delays by evaluating the average delay of 8,596 GPS fixes. The onboard compensation function (see github.com/trichl/WildFiOpenSource [54]) can be used for all tags that integrate a Quectel L70B-M39 GPS module. When using other GPS modules, we recommend checking whether undocumented delays in the time measurements also occur. After synchronisation, we disabled the GPS chip using a microcontroller-operated electronic on-off switch (load switch) to minimise power consumption. We calculated *T*_*off; GPS*_ by comparing the RTC time after synchronisation with the one pulse per second (1PPS) output of a second, independent GPS module (also Quectel L70B-M39). The second, independent GPS module ran continuously for at least 15 min, with a minimum of eight satellites visible. Since the 1PPS output showed no pulse variation after that, we assumed that the second, independent GPS module was suitable as ground truth. We conducted and evaluated 15,453 independent measurements.

### Method B: using WiFi and NTP for onboard UTC time (re)synchronisation

For synchronisation method B (Fig. 2B), we used the WiFi radio on the WildFi tag to connect to local WiFi networks and retrieve time via an internet-accessible NTP server. Previous studies on this tag showed that the synchronisation accuracy depends on the speed of the internet [37]. To address this, we implemented an improved NTP algorithm on the WildFi tag. The algorithm accounts for the transmission delays of NTP messages, thus reducing dependency on the quality of the internet connection. This is especially important when working in the field with slow mobile internet connectivity (e.g., 2G). The improved algorithm assumes a symmetric delay for both sending and receiving NTP data. To conserve energy, only one NTP server request is made (no averaging across multiple NTP requests and servers). WiFi synchronisations require internet access, either via a nearby WiFi router or via a nearby WiFi hotspot generated by a smartphone. We evaluated *T*_*off; WiFi*_ separately based on the internet speed (broadband, mobile LTE, mobile 2G internet). We calculated *T*_*off; WiFi*_ by comparing the time after synchronisation with the one pulse per second (1PPS) output of an external GPS module (Quectel L70B-M39). For maximum time accuracy, the external GPS module ran continuously for at least 15 min, with a minimum of eight satellites visible. We conducted and evaluated 14,792 independent measurements.

### Method C: using wireless proximity messages between tags for onboard relative time (re)synchronisation

For synchronisation method C (Fig. 2C), we used wireless proximity messages that are exchanged between multiple WildFi devices at certain intervals. The signal strength of such proximity messages can be correlated with the distance between tagged animals [55]. We used ESP NOW as the transmission technology, which is based on the WiFi protocol and facilitates wireless communication ranges of up to 200 m [37]. However, the described concept of relative time synchronisation can be implemented using any digital wireless transmission technology (e.g., Bluetooth LE or LoRa). We initially synchronised devices to UTC time using method A (GPS) or B (WiFi). The tags were then programmed to collectively awaken at specific UTC times and exchange proximity messages within an 800-ms-long transmit/receive window (1 message every 100 ms; 8 messages per proximity event). In each proximity message, we transmitted a relative timestamp at ms resolution that marked the time of sending within the 800 ms time window (measured with the microcontroller’s main oscillator; ± 10 ppm drift at room temperature), the current UTC timestamp at second resolution and the UTC timestamp of the last synchronisation. When devices received messages from other devices, they first adjusted the received relative timestamp by subtracting the estimated airtime (5.2 ms) from the received relative timestamp, then compared the received relative timestamp with their own relative timestamp to measure time deviations between devices. If the deviation was greater than 50 ms, devices collectively synchronised their clocks to the clock that had most recently undergone resynchronisation, assuming that clock had a smaller time drift error. The devices that adjusted their clocks then updated the timestamp of their last synchronisation, ensuring that the collectively trusted time was propagated and distributed within a group of devices, even when they were not in constant contact with each other. To evaluate the relative time differences (*T*_*error; relative*_) between multiple tags, we used custom WiFi base stations based on ESP32 CAM modules capable of sniffing proximity messages from nearby tags. Received proximity messages were stored and annotated on the gateways using a relative timestamp derived from the internal high precision clock of the gateway’s microcontroller (± 10 ppm drift at room temperature). For each message, we subtracted the relative time within the 800 ms transmit/receive window from the relative gateway timestamp to estimate a tag’s starting time of a proximity detection in the time system of the gateway. We then calculated pair-wise the time differences of the starting times between all tags participating in a proximity detection. We could not measure the time differences of the relative times to UTC time because the gateway clocks were also subject to time errors and thus not suitable as UTC ground truth. We assumed that the maximum drift of tags adhered to the RTC specification (max. ± 3 ppm between − 40 and 85 °C). We conducted and evaluated 230 independent measurements. Furthermore, we performed a 16-day-long stationary experiment between two tags, continuously monitoring *T*_*error; relative*_. To increase the temporal drift, one tag was placed outdoors (measured temperatures between − 7 and 15 °C) and one tag in the laboratory (measured temperatures between 13 and 21 °C). The tags were programmed to exchange messages every full UTC hour.

### Storing and maintaining onboard time between synchronisations

Time of the RTC on the WildFi devices can be set by the microcontroller via an I2C command with a resolution of full seconds. When setting the time, milliseconds are automatically reset to zero. After receiving time from GPS, WiFi or proximity messages, we programmed the microcontroller to wait with a high accuracy timer derived from the microcontroller’s main oscillator (± 10 ppm drift at room temperature) until the next full UTC second. The time on the RTC was then set via a 9-byte I2C command at 400 kHz from the microcontroller, which added a negligible timing error of 0.18 ms. Between synchronisation events, the RTC drifted within known specifications (max. ± 3 ppm between − 40 and 85 °C). We measured the average power consumption of setting the RTC (*P*_*sync; RTC*_) with an Otii Arc measurement unit set at 3.75 V. When time-annotating recorded sensor data, we retrieved the current UTC time from the RTC via an I2C command after reading the sensor data. We compressed the UTC timestamp, with 10 ms resolution, into 5 bytes (4 bytes for seconds since 1st of January 1970 [standard UNIX timestamp] and 1 byte for milliseconds annotation at 10 ms resolution). This compressed timestamp is stored alongside the sensor data. For free-running sensors (e.g., accelerometers with internal memories), we annotated the end of each data burst with the 5-byte timestamp from the RTC.

### Case study design on Egyptian fruit bats

We deployed WildFi tags with GPS add-on boards on 99 Egyptian fruit bats that roosted together in a cave near Pegeia, Cyprus, between the 27th of January 2023 and the 6th of February 2023 as part of a larger behavioural study. Each tag was powered by a 100 mAh LiPo battery (Axiss Technology Corp., 2.4 g) and housed in heat-shrink tubing (adhesive lined 18 mm Polyolefin, 2.2 g). Tags (7.7 g total mass) were securely affixed to the back of each bat using non-permanent Montreal Ostomy Osto Bond glue. The tags usually fell off after 3 to 10 days. We configured the tags to record GPS, proximity, an 800-ms-long 3-axis 50 Hz burst of acceleration data, temperature, humidity, and barometric pressure every full five UTC minutes. One day prior to deployment, all devices underwent time synchronisation via method A (GPS). Four independent WiFi gateways, powered by 27,000 mAh USB power banks, were positioned within the cave. The gateways ran for 10 days before battery depletion. Throughout the experiment, the tags were programmed to resynchronise via method A (GPS) when outside the cave and method C (wireless proximity messages) when inside the cave, where GPS signals were obstructed. To determine whether a bat was inside or outside the cave, the tags checked whether a gateway was seen during proximity detection. If so, GPS was not activated. This strategy helped to significantly conserve energy on the tags. All data were downloaded wirelessly from the tags to the gateways, so that the bats did not have to be recaptured. The gateways allowed us to evaluate the relative time differences between tags during the entire duration of tag deployment. We evaluated the relative time errors *T*_*error; relative*_*(t)* between tags based on the algorithm previously described for method C.

## Results

### UTC and relative accuracy of onboard synchronisation methods in stationary tests

**Fig. 3:**
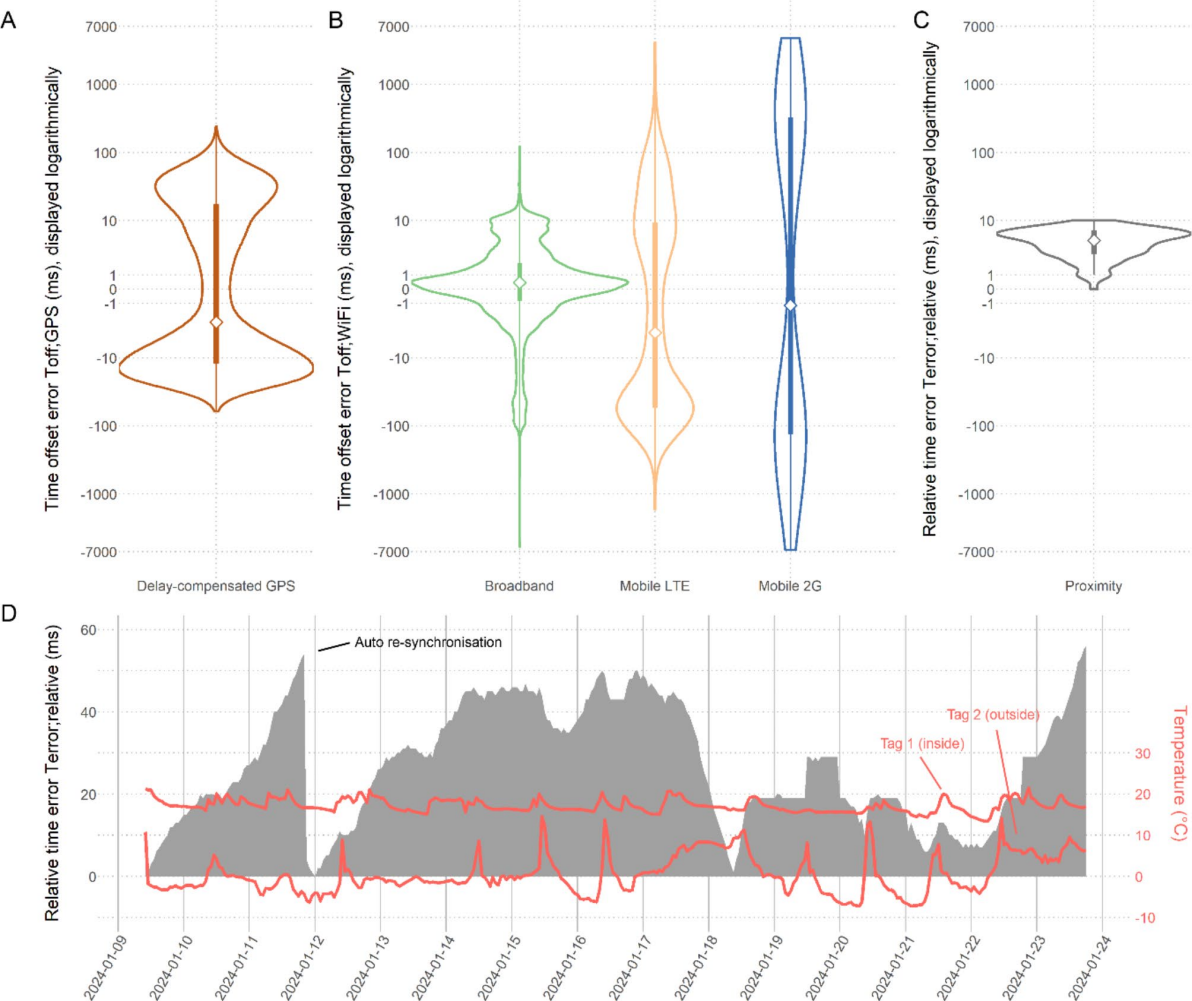
Achieved time accuracies with optimised methods for automatic time synchronisation on bio-loggers in stationary experiments (GPS [**A**], WiFi and NTP [**B**], wireless proximity messages [**C**-**D**]). The achieved accuracies with WiFi (**B**) are separated by the internet speed (broadband, mobile LTE, mobile 2G). During the 16-day-long relative time synchronisation experiment between two tags (one positioned outside, the other inside), an automatic resynchronisation was triggered because the tags measured a relative time difference of more than 50 ms onboard (**D**). The tag temperatures were measured with an onboard temperature sensor (Bosch Sensortec BME680)

In the stationary tests of the synchronisation methods, we measured a median error *T*_*off; GPS*_ of 2.72 ms (MAD 12.47; *N* = 15,453) with method A (Fig. 3A). For method B we measured median errors *T*_*off; WiFi*_ of 0.44 ms (MAD 1.32; *N* = 13,767) when using broadband internet, 4.1 ms (MAD 37.12; *N* = 853) when using mobile LTE internet, and 1.32 ms (MAD 199.76; *N* = 172) when using mobile 2G internet (Fig. 3B). The overall measured median error *T*_*off; WiFi*_ equalled to 0.43 ms (MAD 1.51; *N* = 14,792), but the result is statistically dominated by the large N of the broadband tests. For method C we measured a relative time error *T*_*error; relative*_ between two tags of median 5 ms (MAD 2; *N* = 230) immediately after resynchronisation (Fig. 3C). In the 16-day stationary experiment we measured a median relative time error *T*_*error; relative*_ of 28 ms (MAD 13; *N* = 344), and a final relative time error of 56 ms after the experiment (auto-resynchronisation once on day three) (Fig. 3D).

### Relative time accuracy of combined onboard synchronisation methods on Egyptian fruit bats

**Fig. 4:**
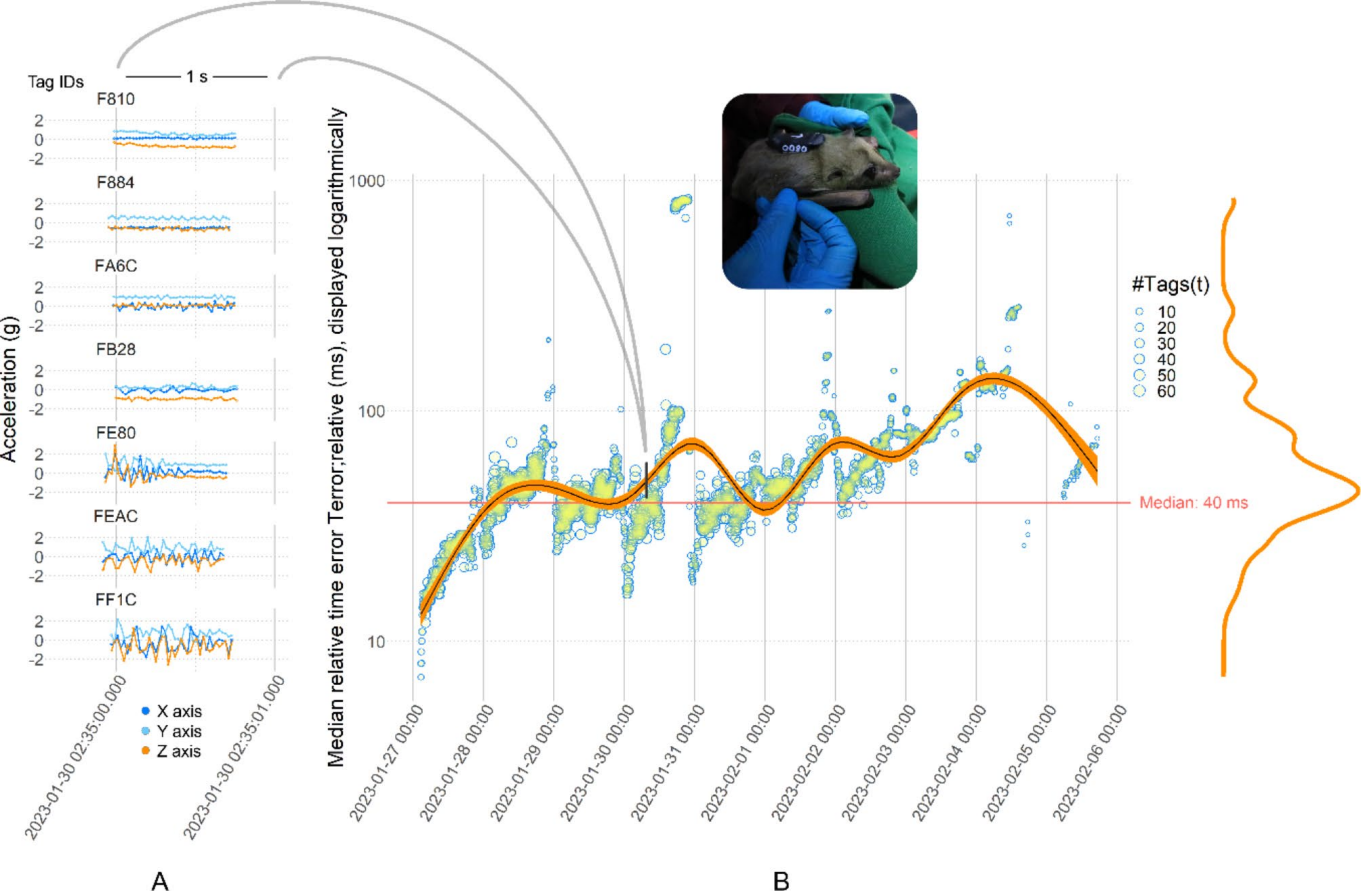
Time synchronisation accuracies achieved on 99 bio-loggers attached to Egyptian fruit bats. Time differences were calculated using the time of arrival of proximity messages on data gateways placed inside the bat cave (**B**). Precise time synchronisation permitted the alignment of 800-ms-long 50 Hz acceleration bursts across multiple tags (exemplified in A; bursts are drawn with real time offsets). This capability facilitates the analysis of collective bat behaviour at an exceptionally proximate temporal resolution

After deployment, the tags collected data up to 10 days before battery depletion. The median relative time difference *T*_*error; relative*_*(t)* between tags during the entire duration of deployment equalled 40 ms (MAD 29, *N* = 933,411) (Fig. 4B). Figure 4A exemplifies how our methods automatically time-aligned 800-ms-long 50 Hz acceleration bursts that were recorded every full five UTC minutes. All recorded sensor data (e.g., temperatures) were timestamped and thus also synchronised at a median of 40 ms.

### Energy considerations of onboard time synchronisation

For updating the real time clock after receiving a new time via method A, B or C, we measured an average energy consumption of 6.5 µWh (*P*_*sync; RTC*_), which included the waiting time until the next full second using the high precision timer of the ESP32 microcontroller. If GPS data are recorded as part of a study, then Method A (GPS) only requires additional energy to set the real time clock (*P*_*sync; GPS*_*= P*_*sync; RTC*_). Method B (WiFi) requires additional energy to establish a connection and retrieve time via NTP, but also allows for wireless data transmissions. Previous research has shown that the average power consumption (*P*_*sync; WiFi*_) was 412.5 µWh, including the energy needed to set the real time clock (*P*_*sync; RTC*_) [37]. If tags are already recording proximity data as part of a study, method C (wireless proximity messages) only requires additional energy to set the real time clock (*P*_*sync; relative*_*= P*_*sync; RTC*_). Keeping time between synchronisations required 720 nW (power consumption of the Micro Crystal RV-8803-C7 RTC).

### Comparison with existing time synchronisation solutions in post processing

To our knowledge, the current literature is devoid of information regarding onboard time accuracies of bio-logging devices. When non-temperature-compensated clocks from common microcontrollers are used to annotate bio-logging data, time errors can reach ± 4.3 days after one month of logging (see Table 1). So far, such time errors in data have only been addressed and corrected in post processing (e.g., by linear regression [15, 56]), but never on the devices themselves. Most post processing methods are either complex or assume a linear drift in time, potentially resulting in undefined time errors. Importantly, post processing corrections do not allow devices to perform precisely timed actions (e.g., communications between tags or other systems). A 2019 study found average discrepancies of up to 28 s between acceleration and GPS data (recorded on the same device) after 9 days [39]. Similarly, in another study, which simultaneously recorded acceleration and audio data on Canada lynx, the authors estimated the time error between devices after post processing to be ± 2 min [56]. A study on snowshoe hares achieved time synchronisation of ± 30 s on average between acceleration and audio data recorded in parallel for 62 days, with post processing [15]. In a 2024 study, multiple tags were attached to different parts of a fish’s body and the measured average relative time drift between devices was ± 0.5 s after 24 h (~ 6 ppm) [24]. A recent proximity logger design demonstrated how the long-range transmission of relative timestamps can be used to compensate for clock drift, but with unspecified accuracies [26]. In comparison, our onboard synchronisation methods can be used on any type of sensor data and enable a permanent UTC time accuracy (*T*_*off; GPS+WiFi*_*+ T*_*drift*_) of ≤ 60 (185) ms in 95% of cases over a wide temperature range between 0 and 50 °C when resynchronising only once per hour (day) (Table 2).

**Table 2:** The resulting permanently achievable maximum time errors compared to UTC time when using GPS and/or WiFi as synchronisation methods

Ambient temperature range (°C)	Onboard resynchronisation interval (WiFi / GPS)	Maximum T_error_ (75% quantile) (ms)	Maximum T_error_ (85% quantile) (ms)	Maximum T_error_ (95% quantile) (ms)
0 to 50	10 min	± 18	± 27	± 56
-40 to 85	10 min	± 19	± 28	± 57
0 to 50	60 min	± 23	± 31	± 60
-40 to 85	60 min	± 28	± 37	± 66
0 to 50	24 h	± 147	± 156	± 185
-40 to 85	24 h	± 277	± 286	± 315

## Discussion

The field of bio-logging is growing rapidly, and with it the variety, amount, and density of ecological data collected “at the same time”. Time errors in bio-logging data are currently either overlooked, ignored or tackled in post processing. Post processing methods are complex and error prone, especially for datasets recorded over long periods of time [15, 57]. There is a pressing need for new methods to enhance temporal accuracies directly at the data source, i.e., on board animal-borne devices. Accurate timestamps are essential for matching multiple data streams (e.g., for training machine learning algorithms to classify behaviour), for allowing event-based controls (e.g., timed remote data downloads to overpassing satellites or drones [11, 28, 29]), and for reducing energy-intensive transmit/receive windows (e.g., with proximity tags). In this study, we first developed an error model for time measurements within bio-loggers collecting ecological data. We then used the open source WildFi tag to combine a temperature-compensated RTC with three optimised methods for obtaining UTC and/or relative time (GPS, WiFi, proximity messages), and measured time accuracies in off-animal tests. For GPS we implemented a delay compensation and measured median offsets (*T*_*off; GPS*_) of 2.72 ms (MAD 12.47). With WiFi and an improved onboard NTP algorithm, we measured median offsets (*T*_*off; WiFi*_) of 0.43 ms (MAD 1.51). Slow internet had a negative impact on the median time accuracy (*T*_*off; WiFi;2G*_ = 1.32 ms [MAD 199.76]). Finally, we implemented a method for relative time synchronisation between a group of tags based on the exchange of wireless proximity messages. This method is adaptable to any wireless two-way digital communication system (e.g., Bluetooth LE). Here, we were able to achieve a median relative time accuracy (*T*_*error; relative*_) between tags of 5 ms (MAD 2) immediately after synchronisation and consistently under 56 ms over the course of 16 days. We combined multiple methods within a case study on 99 Egyptian fruit bats, that successfully kept tags time-synchronised to a median of 40 ms (MAD 29) over the entire 10-day tag deployment. This advancement permits comparison of simultaneous movement patterns of individuals at a fraction of a second, opening up new possibilities for analyses of animal behaviour. Our results outperform all existing post processing methods for time alignments, demonstrating that milliseconds-accurate time annotation of any type of bio-logging data can be achieved with current technology, even in long-term experiments, through smart (re)synchronisation software and optimised components.

Regardless of bio-logger type, we recommend tag users to ask device manufacturers for specifications of the internal clocks used for annotating data with time, especially when onboard synchronisation is not available (e.g., tags without GPS, like micro-sized acceleration, temperature, light and/or pressure loggers [9, 42, 43]). For tag developers, we strongly recommend integrating a temperature-compensated RTC on devices and using it as central source for annotating multi-sensor data with time. Modern RTC chips are small and add just a few nanowatts to the overall device power consumption. Temperature compensation is particularly important for devices carried by free-roaming animals, since there are often large temperature fluctuations in the environments they inhabit [48], leading to large time drifts. Even with the integration of low-drifting RTCs, we recommend resynchronising tags onboard (methods A - C) at least once daily to achieve a guaranteed sub-second time accuracy. Furthermore, we recommend testing the time accuracy of GPS modules, as we found undocumented delays caused by position processing and message delivery. 1PPS outputs from GPS modules appeared to be more accurate time sources. We note that when comparing tag data with external data, the timing accuracy of both systems should be considered. Therefore, we would also like to encourage manufacturers of off-animal devices (e.g., video cameras) to integrate time synchronisation methods and quantifying time errors to improve data comparability among and within systems.

Temporal accuracies of bio-loggers are often mentioned as a limiting factor when integrating multiple data streams, but are rarely quantified [11, 24, 31–33]. Accuracies achieved through post processing methods typically range from several seconds to minutes for multi-day recordings [15, 39, 56]. In contrast, our methods offer long-term time accuracy in the sub-second range. One limitation of our solution is that the WildFi tag can store time with a maximum resolution of 10 ms, which inherently limits data accuracy even with perfect time synchronisation. Although we optimised the NTP algorithm of the WiFi synchronisation method to consider round-trip times of time messages, the speed of the internet still influences the time accuracy. This limitation could be further addressed in future developments.

By accurately aligning the ever-growing amount of data across multiple loggers, sensors, and measurement systems, ecologists can build a more complete picture and deeper understanding of patterns of behaviour and the biological processes at play. Our proposed methods offer a possible solution for fully automating the improvement of time measurements on bio-loggers. We argue that time measurement requires more scientific attention in the bio-logging community and ‘time’ should be treated as a distinct sensor data type, where quantified accuracies and error-reducing methods are necessary to draw robust and reliable conclusions, as is common with spatial data.

## Conclusions

Our study addresses the critical need for accurate timestamps in bio-logging data. We demonstrate the feasibility of long-term sub-second time accuracy across devices through the utilisation of temperature-compensated RTCs alongside three combinable low power synchronisation methods (GPS, WiFi, wireless proximity messages). The proposed methods reduce time errors fully automated on the devices, obviating the need for further corrections during post processing. In stationary tests, we measured median synchronisation accuracies of 2.72 / 0.43 / 5 ms (GPS / WiFi / proximity), showing the validity of our approach. On 99 Egyptian fruit bats, we maintained device synchronisation with a median time error of 40 ms between each other over a period of 10 days. This level of time precision significantly enhances the quality of ecological studies and allows new scientific questions to be asked. We argue that the measurement of time warrants greater scientific attention within the community.

## Data Availability

The stationary experiment data, the case study data on Egyptian fruit bats relevant to the evaluation of time accuracy and the code used in this paper are available at github.com/WildLab/WildFiTimeSyncData or through Zenodo [58]. The complete dataset from the case study on Egyptian fruit bats will be published in the future, as it is part of an ongoing study.

## Abbreviations

1PPS: One pulse per second
2G: Second generation
AI: Artificial intelligence
GPS: Global positioning system
I2C: Inter-IC bus
IMU: Inertial measurement unit
IoT: Internet of Things
LiPo: Lithium-polymer
LoRa: Long range
LTE: Long-term evolution
MAD: Median absolute deviation
NMEA: National Marine Electronics Association
NTP: Network time protocol
OCXO: Oven-controlled oscillator
ppm: Parts per million
RTC: Real time clock
TCXO: Temperature-compensated oscillator
UART: Universal asynchronous receiver transmitter
USB: Universal serial bus
UTC: Universal time coordinated
WiFi: Wireless fidelity
XO: Oscillator
